# Supplementary material for: Micro-structure diffusion scalar measures from reduced MRI acquisitions
Source: PLoS One. 2020 Mar 9;15(3):e0229526. doi: 10.1371/journal.pone.0229526 (PMC7062271; doi:10.1371/journal.pone.0229526)
Supplement: Attachment — Submitted filename: Response to reviewers.pdf [file pone.0229526.s001.pdf]

UNIVERSIDAD DE VALLADOLID

Antonio Tristán Vega, Ph.D.
Dpt. Mechanical Engineering
Universidad de Valladolid. Spain

Rebuttal letter. Response to reviewers

PONE-D-19-27308

“Micro-structure diffusion scalar measures from reduced MRI acquisitions”

First of all, we’d like to thank the Reviewers for their comments, which we have found both constructive and useful. In what follows we try to address all the concerns they mention in their reports.

Before that, we point out here we have detected a **minor erratum** in the derivation of one of the measures (RTPP) while preparing the second version of the manuscript: concretely, the formula for RTPP (eqs. (8) and (12), second row in Table 1) was wrongly divided by a factor 2 in the former version. Note this has a null impact in all the experiments throughout the paper, since a constant scale-shift will not change the correlation coefficients or the classification capability of RTPP. However, we have accordingly corrected whatever figures/tables/results in the paper affected by this erratum. These changes have been highlighted in the marked copy of the manuscript in color yellow.

Besides, we would like to point out here that all the Figures included in the paper were specifically designed for it and have not been published elsewhere, so no copyright issues apply.

As for the changes arising from the comments by Reviewer 1, they have been highlighted in the marked copy in color blue. Those changes made in response to the comments by Reviewer 2 are highlighted in color green.

Finally, the particular concerns by the Reviewers are answered below one by one.

Reviewer #1: The purpose of this work is to present a method for the estimation of the microstructural scale, e.g. RTOP, RTPP, and RTAP from single-shell acquisitions. The paper presents a simplified formulation to calculate these scales with the assumption that the diffusion anisotropy is roughly independent of the radial direction. The proposed work proves its usability in the different acquisitions. The manuscript is concise and well explained and organized. I have a few comments on the manuscript:

R1.1. "...assuming the diffusion anisotropy is roughly independent of the radial direction.", Please have an explaining of the rationality of this hypothesis for clinical applications. Also, what are the special requirements for the acquisition scheme?

As stated on the paper, this is a common assumption in some diffusion modalities, like HARDI. It cannot be sustained for the whole range of b-values (i.e. for the whole q-space), but it can be assured if we consider a small interval around a given b-value. We are aware that, on one hand, we are losing some information here but, on the other hand, the gain has a great implication on real application. Buy assuming independency with the radial direction, we are able to formulate the proposed measures using just one single shell. As a consequence, the acquisition time in the scanner is reduced and the measures can be used in real clinical

studies, using data acquired in reasonable time. The good thing here is that no special requirements are needed for the acquisition scheme. At least, nothing far from common acquisitions. We recommend a b-value higher than 2,000 s/mm^2, since the effects we want to measure are stronger in that range. We also recommend a uniform sampling of the sphere. Anyway, the standard acquisition for HARDI data, or for the outer shell of an acquisition intended for Kurtosis imaging or CHARMED.

R1.2. Please also give some results to explain the impact of the number of gradient samples (or angular resolution) on the results.

We agree that, since we claim AMURA to be suitable for acquisition protocols based on reduced acquisitions, the impact of the actual number of gradients in the results is an interesting study. Accordingly, we have re-written the corresponding section, now entitled "Variability of apparent measures depending on the acquisition parameters", to include this additional study (together with the previous one with the impact of the b-value). To do so, Fig. 6 have been upgraded with additional measure-versus-number of gradients curves.

We thank the reviewer for suggesting this experiment. Results have shown that the proposed measures are really robust to the change in the number of gradient directions.

R1.3. Note that the experiment implemented in higher b-value on the result, e.g. Figure.1 and Table.3. What happened with b=1000?

The measures considered in this paper (RTOP, RTPP and RTAP) are known to describe processes that need higher b-values to occur. In particular, it is usually recommended to work over 2,000 s/mm^2 in order to properly estimate them. For lower values, we could also obtain some measure, but will not properly describe the desired measures. Actually, results for b=1,000 s/mm^2 differ from the rest. This is why we have decided to leave them aside.

R1.4. Please clarify the definition of the correlation coefficient in Table.3.

We have added the new equation (14), together with corresponding explanations, to precisely define this parameter.

R1.5. In Figure.1, the RTPP calculated by AMURA seems to be significantly different from the results from other others. which is obviously cleaner. It seems to be related to the hypothesis that diffusion anisotropy is roughly independent of the radial direction. But it's still unclear whether this kind of change is an improvement. So it is better to show the correlation of RTPP with MD or RD.

It is true that RTPP is noisier than RTOP and RTAP because it is computed as a maximum operator, as opposed to RTOP and RTAP, which are computed as averages. Of course, since AMURA rely on a constrained model, they have to estimate fewer degrees of freedom than EAP-based techniques, yielding to a "cleaner" (less noisy) estimation of RTPP.

The Reviewer is right that a "cleaner" image is not necessarily an improvement, since it might be blurring out relevant information. Indeed, this is exactly what we are trying to test throughout the experiments in the paper: is AMURA able to preserve the anatomical information of interest even when it "smooths" the data due to the use of a radial model? It seems so, at least for the kind of clinical data sets used in the paper.

With regard to the suggestion of correlating RTPP with MD or RD, note they are somehow opposed. The Reviewer may check in the Appendix that the RTPP for the tensor model basically reduces to the inverse of (the square root of) the largest eigenvalue λ_1. Both the MD and the RD are directly proportional to the eigenvalues (either their average or the largest one), so that the RTPP and the MD/RD will correlate

negatively. For example, if we pay attention to the CSF, where unrestricted diffusion prevails, the MD/RD will exhibit the largest values throughout the brain, meanwhile RTPP will be very small.

Of course, we could correlate RTPP with the inverse of the RD or its squared root, and we could expect strong correlations. In the same way, RTAP and RTOP will have positive correlations with the FA, as it is suggested by Figs. 1, 6, and 8 of the new version. However, the aim of this paper is precisely to check if AMURA can infer anatomical information beyond the tensor model from one single shell, so we still think the comparison to perform is against EAP-based techniques.

Finally, the Reviewer made us pay new attention to the structure of RTPP maps that, as stated before, will resemble the inverse of the MD/RD, as opposed to RTOP and RTAP, which resemble FA/RA maps. Indeed, for the tensor model, it is easy to check that RTOP = RTAP·RTPP, meaning that RTAP and RTPP are somehow complimentary (this detail has been included in the new version of the Appendix).

This fact also explains why in Figs. 6 and 8 (in the new version) the plots for RTPP cross each other between clusters with different FA values: since RTPP is more related to the MD/RD than to the FA/RA, there is no reason to expect that clusters ordered as a function of their FA will preserve the same order with respect to their RTPP. A brief discussion on this topic has been included in the paper, in the section now entitled "Variability of apparent measures depending on the acquisition parameters". Besides, the newly introduced Fig. 7 reinforces this argument by repeating the experiment with a different clustering not based on the FA.

Reviewer #2: In this paper, the authors propose a set of novel diffusion indices, called apparent measures using reduced acquisitions (AMURA), which can mimic the EPA-derived indices, but require only single-shell dMRI data. AMURA are based on the observation that EAP-derived indices are radial integrations of a set of measures at different shells. Based on this observation, the authors simplify the acquisition to a single-shell case and propose a set of single-shell measures to mimic EPA-derived indices, including RTOP, RTPP, and RTAP. The authors first show the analytical solutions for AMURA and then provide detailed numerical implementations by using spherical harmonics. AMURA is evaluated using a normal dataset, HCP, and a patient dataset, PPD. Three baseline methods, including RBF, MAPL, and MAP-MRI, are involved in the evaluation. Extensive experimental results demonstrate the effectiveness of AMURA, both qualitatively and quantitatively.

The paper is written well and motivation is clearly stated. AMURA are novel indices, which allow the study of brain disorders using EPA-like indices and clinical dMRI data. Regarding the proposed method, I have several minor concerns.

R2.1. AMURA change at different b-values. Is there an optimal b-value for AMURA? For instance, which b-value gives the best AMURA that are most similar to the EAP-derived indices?

This question is not easy to answer. First, we cannot talk about "the apparent measure that is most similar to EAP-derived measures", since EAP-derived measures show themselves a strong dependency with the acquisition parameters (see Fig. 8 in the new version). Second, at the sight of Figs. 6 and 7 in the new version, AMURA seem to preserve a sort of "ordering prevalence" as the b-value increases: i.e., the value of AMURA varies as the b-value changes, but the relative differences between clusters remain, so that the discriminant power of AMURA seems also the same for all b-values.

If we had to give a short answer to this question, we'd say that the optimal b-value for AMURA is, for a given application (i.e. a given classification problem of white matter voxels) and a given data set, that providing the highest inter-class separability.

Yet, one of the main ideas of the paper is that AMURA can be used over already existing data sets to probe new information (this was already stated in the previous version of the paper). In case several shells are available in one such data set, AMURA can be trivially extended to gather all the information together by

simply including the b-values of each shell in the mono-exponential model, hence there is no need to actually choose one of the available shells. This brief discussion has also been included in the paper (discussion and conclusions section).

R2.2. Page 10: A threshold is selected in the valley right after the main lobe in each case (for RTOP: 2 · 10^6 mm^−3; for RTPP: 90 mm^−1; for RTAP: 1.5 · 10^4 mm^−2). Please clarify whether all comparison methods share the same set of thresholds, or not.

Indeed, these "fixed" thresholds are only used to obtain a "bronze standard" from MAPL, i.e., to obtain to disjoint classes of voxels. In order to compare the other methods to the bronze standard, we do not use one single threshold for each measure and estimation technique. Instead, we use the ROC curve method:

- Choose one measure (e.g. RTOP) and one method (e.g. RBF).
- Compute the minimum value of RTOP obtained with RBF, say min, and the maximum, say MAX.
- Consider a sufficiently large number of thresholds "th" ranging in [min,MAX].
- For each such threshold, assign those voxels with RBF-RTOP<th to class1, and those with RBF-RTOP>th to class 2.
- Compare this classification to the "bronze standard" obtained with fixed thresholds over MAPL-RTOP. "Actual" class1 voxels classified as class2 are false positives. "Actual" class2 voxels classified as class1 are false negatives.
- Plot a parametric curve (the parameter here is the threshold "th") of false positives vs. false negatives. This is the ROC curve.

This is a standard methodology in pattern recognition and classification problems. However, we have re-written the description of the method trying to clarify this point.

R2.3. The experimental results are convincing and sufficiently demonstrate the effectiveness of AMURA. However, I am wondering whether synthetic data experiments are useful or not since we do not have ground truth EAP measures in real data experiments.

We share the concern of the Reviewer about using synthetic data for the validation of this kind of methodologies. Indeed, all the experiments included in the paper make use of real data sets obtained from public databases (hence the term "bronze standard" for the experiment described in Fig. 2).

Though we devised a series of experiments with synthetic data for a preliminary version of the manuscript (which can be accessed at BioArxive: https://www.biorxiv.org/content/10.1101/772897v1), we decided not to include them in the final version because they did not seem conclusive.

Note AMURA aims at describing micro-structural properties of the tissues in a consistent way with MAPL, MAPMRI or RBF. However, synthetically generating signals that can represent such properties in a realistic environment is not a trivial task. It is easy to simulate a signal coming from crossing fibers or a mixture of free and axon-confined water, but modulating the signal with the shape and size of cellular pores in a realistic manner is far more difficult. We tried to do so by simulating voxels with mixtures of cylindrical, elliptical, or spherical pores of different sizes mixed up with free water at a given value of the FA (somehow similar to the experiments in Figs. 6, 7, and 8), but we ended up with one of these two scenarios:

- The two groups designed were so much different that almost any diffusion-related measure (multi-shell, tensor, or AMURA) was able to trivially distinguish between them.
- The two groups designed were almost identical and not a single measure (multi-shell, tensor, or AMURA) was able to consistently distinguish between them.

Since we were not able to design any reasonable experiment that reported consistent results, we decided to restrict the evaluation to real data, being conscious of the limitations (i.e. the lack of a golden standard) this methodology imposes.
